# Developing compatibility biomass model based on UAV LiDAR data of Chinese fir (*Cunninghamia lanceolata*) in Southern China

**DOI:** 10.3389/fpls.2025.1520666

**Published:** 2025-09-26

**Authors:** Zheyuan Wu, Dongbo Xie, Ziyang Liu, Qiao Chen, Qiaolin Ye, Jinsheng Ye, Qiulai Wang, Xingyong Liao, Yongjun Wang, Ram P. Sharma, Liyong Fu

**Affiliations:** ^1^ Research Institute of Forest Resource Information Techniques, Chinese Academy of Forestry, Beijing, China; ^2^ College of Information Science and Technology & Artificial Intelligence, State Key Laboratory of Tree Genetics and Breeding, Co-Innovation Center for Sustainable Forestry in Southern China, Nanjing Forestry University, Nanjing, China; ^3^ Guangdong Forestry Survey and Planning Institute, Guangzhou, China; ^4^ Chengdu Academy of Agriculture and Forestry Sciences, Chengdu, China; ^5^ Institute of Forestry, Tribhuvan University, Kathmandu, Nepal

**Keywords:** biomass model, dummy variable model, compatibility model, growth and development stage, Cunninghamia lanceolata

## Abstract

Chinese fir (*Cunninghamia lanceolata*) is a key native tree species in southern China. Accurate estimation of above-ground biomass and its distribution is essential for the sustainable use of Chinese fir forests. UAV-based high-density point clouds and high-resolution spectral data provide critical remote sensing for detailed 3D tree structure analysis. This study aimed to explore the aboveground biomass allocation characteristics across the different growth stages of Chinese fir and to develop accurate biomass models. Measurements of 20,836 Chinese fir trees were used for the purpose. Through the comparative analysis of four basic models, the Power Function model was identified as the optimal one, particularly excelling in fitting the accuracy for stem and bark biomass. To further enhance the model’s fitting performance, age groups were introduced into the dummy model, categorizing the Chinese fir forests into the five distinct growth stages. Results showed age groups used as dummy variables led to an average increase in *R²* by 2.6%. The fitting accuracy for bark and branch biomass saw the most significant improvements, with increases in *R²* by 4.2% and 3.1%. To address the inconsistency between the sum of individual biomass components and total biomass, we employed a seemingly unrelated regression (SUR) model. Even though fitting accuracy for individual tree components decreased by an average of 2.5%, from a practical perspective SUR model would be more suitable for understanding the interrelationships between different components. These findings offer robust support for accurately estimating the aboveground biomass in Chinese fir forests across different growth stages.

## 1 Introduction

Forest ecosystems are the pillars of terrestrial ecosystems (Field and Raupach, 2012), occupying a critical role in the Earth’s ecological system and playing a significant regulatory function (Subati and Jia, 2021). As fundamental attributes of forest ecosystems, estimation of forest biomass has become key issues in forest research and forestry applications (Lieth and Whittaker, 2012). Accurate forest biomass models not only form the basis for evaluating forest carbon sequestration capacity but also provide critical scientific support for carbon trading mechanisms and climate change policy development (Ma et al., 2024; Zhang J. et al., 2019). However, current biomass estimation methods still face various technical challenges, such as difficulties in field data collection, inefficiency of traditional biomass estimation techniques, significant measurement errors, and limited generalizability of biomass models across different regions and tree species (Weiskittel et al., 2015). These challenges can lead to uncertainties in forest carbon stock estimates, subsequently affecting the accuracy of ecological management decisions (Holdaway et al., 2014). Therefore, developing more efficient and accurate biomass estimation models and improving existing technologies have become pressing priorities in forest carbon sink research (Holdaway et al., 2014).Estimating aboveground biomass, as a crucial component of forest ecosystems, holds immense importance within forest science and forestry practices. This is particularly significant for carbon cycle research, ecosystem service evaluation, and sustainable forest management (He et al., 2013). However, there is considerable uncertainty in estimating forest carbon sink, which can be mitigated by precise estimation of forest biomass (Fu et al., 2022). Therefore, accurately estimating forest biomass is of paramount importance, which can be done using direct and indirect methods. The former method is the most accurate, but time-consuming, labor-intensive, and highly destructive, whereas later method involves developing biomass models (Wang et al., 2018a). Constructing biomass models, as the primary approach for estimating forest biomass, represent an effective and relatively accurate method of investigation. With the advancement of mathematical modeling techniques, the methods for developing tree branch biomass models have evolved from simple least squares regression to more sophisticated and precise modeling, including constructing compatible models that account for measurement errors (Zeng and Tang, 2010b; Zhang et al., 2016). The advanced modeling encompasses seemingly unrelated regression models (SUR), linear or nonlinear joint estimation models (Tang et al., 2000), dummy variable models (Zeng et al., 2011), and mixed-effects models (Fu et al., 2012, 2013, 2016). These models, when applied to tree-scale data, better reflect the biomass distribution across different parts of the tree, providing scientific support for carbon storage and growth analysis at the individual tree level.

When applied to tree-scale data, better reflects the biomass distribution across different parts of the tree, providing scientific support for carbon storage and growth analysis at the individual tree level. Given the critical importance of accurate biomass estimation, the evolution from simple regression methods to more complex and precise models underscore the need for continual refinement in this area. As forest ecosystems continue to play a vital role in global carbon cycles and ecosystem services, the development and application of these sophisticated biomass models are essential for advancing our understanding and management of forest resources. However, traditional manual survey techniques for obtaining individual tree biomass parameters are time-consuming, labor-intensive, inefficient, and lack timeliness, making them increasingly inadequate for the precise monitoring of digitized forest resources under new conditions (Wan et al., 2021). In contrast to conventional remote sensing, Unmanned Aerial Vehicle Light Detection and Ranging (UAV LiDAR) technology can penetrate forest canopies, capturing three-dimensional structural information of forests by emitting laser pulses and receiving reflected signals. This technology provides complete data from the tree top to the base, which is crucial for estimating tree volume and biomass (Wallace et al., 2012; Ghanbari Parmehr and Amati, 2021). This high-precision remote sensing technology captures detailed three-dimensional structures of both the ground surface and vegetation (Dandois and Ellis, 2010), acquiring critical details such as tree height, canopy density, and terrain-key parameters for biomass estimation (Zhang D, et al., 2019). Consequently, UAV LiDAR technology is increasingly used to accurately measure the biomass of individual trees and their branches. This study explores the use of UAV LiDAR data to construct an additive biomass model for Chinese fir forests across the full growth cycle in Guangdong. The application of this technology not only enhances the efficiency and accuracy of forestry resource surveys, but also provides reliable foundational data for forest carbon storage assessment and biodiversity conservation, thereby laying a solid foundation for long-term monitoring and sustainable management of forest ecosystems. This approach can subsequently be extended to biomass estimation across entire forest stands (Fang et al., 2015).

Chinese fir (*Cunninghamia lanceolata*) is recognized for its high-quality timber and significant economic value in forest management, making it one of the most important economic tree species in subtropical regions. Moreover, Chinese fir plays an indispensable role in forest ecosystems (Yu et al., 2010). Its aboveground biomass constitutes a major component of forest carbon storage, holding significant implications for global carbon cycle research and climate change mitigation. The biomass of Chinese fir not only affects the carbon storage capacity of forests but is also closely linked to biodiversity and soil quality. Therefore, studying the adaptability and biomass allocation strategies of Chinese fir across different growth stages has become a focal point in global forestry science research (Dandois and Ellis, 2010). There are notable differences in the biomass allocation strategies of Chinese fir at various growth stages (Li et al., 2022). As the growth stages advance, particularly during the young and middle-aged phases, the growth rate of Chinese fir accelerates significantly, with biomass increases primarily concentrated in the stem and branch components. The biomass growth during this stage is especially pronounced, laying a crucial foundation for subsequent carbon storage. Accurately assessing the aboveground biomass of Chinese fir at different developmental stages facilitates more precise calculations of the forest’s carbon sequestration capacity but also provides scientific evidence for forest management and ecological restoration. Analyzing the biomass structure of various branches of Chinese fir at different growth stages allows for a deeper understanding of the role of aboveground biomass in revealing the mechanisms by which plants adapt to environmental changes (Peng et al., 2017; Sun et al., 2022). Therefore, in-depth research into the biomass allocation and growth patterns of Chinese fir not only enhances the forest’s carbon storage capacity but also promotes the development of sustainable forestry, supporting the achievement of both ecological and economic benefits.

Forest biomass can be divided into aboveground and belowground biomass, with the aboveground portion further subdivided into four components: trunk, bark, branches, and leaves. Previous studies have often modeled these components independently to achieve the required precision (Jingyang et al., 2016; Lun et al., 2018). However, in forest biomass estimation, data are frequently subject to various errors, leading to discrepancies where the sum of the aboveground biomass components does not equal the total aboveground biomass. If traditional models fail to address these errors adequately, the resulting estimates may be biased, and their statistical power may diminish. In contrast, systems of simultaneous equations can effectively utilize the information within the data, optimizing statistical efficiency to address this issue. To ensure that the predicted sum of biomass components equals the total aboveground biomass (Wang et al., 2017) and to explore the growth conditions of Chinese fir forests at different developmental stages, this study employs nonlinear models, dummy variables, and systems of simultaneous equations. These models link the total aboveground biomass of Chinese fir and its components (including bark, stem, branches, and leaves) with predictive factors derived from LiDAR point cloud data, such as LiDAR tree height and LiDAR crown diameter. For each component, an equation is established, and these equations are combined into a system of simultaneous equations. This approach ensures the integration and consistency of all biomass components, providing a method for a more accurate and comprehensive assessment of forest biomass (Parresol, 1999).

This study focuses on utilizing UAV LiDAR data to obtain high-precision measured data and ground survey data of Chinese fir forests in Guangdong and establishes a systematic and robust modeling system that includes nonlinear models, dummy variable models, and systems of simultaneous equations. The aim is to accurately estimate the aboveground biomass of Chinese fir at different growth stages (including stem wood, bark, branches, and leaves), thereby improving the accuracy of forest biomass estimation and ensuring the structural consistency of biomass components. Unlike traditional biomass estimation methods that rely on destructive sampling and empirical models, this study leverages cutting-edge UAV LiDAR technology to enhance efficiency, accuracy, and scalability in biomass assessment. By integrating advanced modeling techniques with remote sensing data, our research not only provides a scientific basis for precise forest carbon estimation but also supports large-scale forest monitoring and sustainable forest management. The findings of this study are expected to contribute to climate change mitigation efforts by improving the accuracy of carbon stock assessments and providing essential data for carbon trading policies. The primary objectives of this study are: 1) to accurately estimate the biomass of individual tree components in Chinese fir forests in Guangdong using UAV LiDAR data; 2) to construct biomass models for Chinese fir at different growth stages and explore the biomass allocation characteristics across different growth cycles; 3) to ensure the consistency between the biomass of different components by constructing a compatibility model, providing a reliable basis for forest carbon assessment and sustainable management.

## 2 Materials and methods

### 2.1 Methodological framework

**Figure 1:**
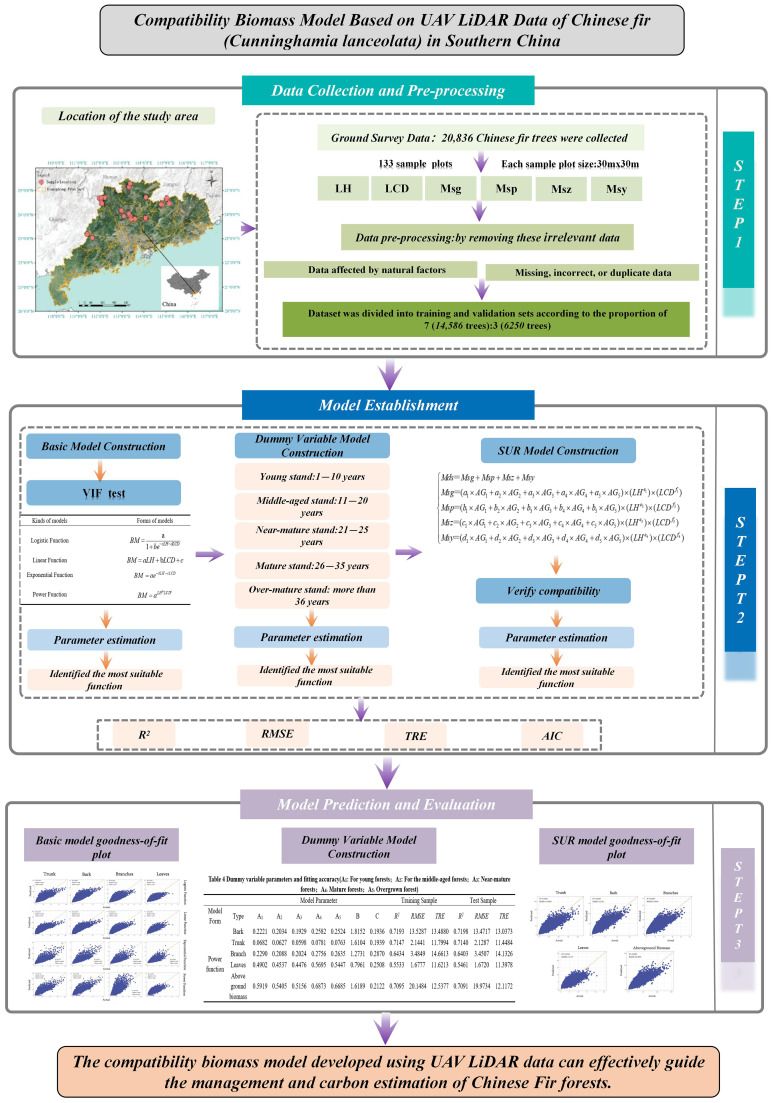
Framework.

**Figure 1** presents the flowchart of the methodology used in this study. We developed a compatibility biomass model for the aboveground branches of Chinese Fir (*Cunninghamia lanceolata*) using LiDAR measurement data from 133 plantation sample plots in Guangdong Province, totaling 20,836 trees. First, we used LiDAR-derived tree height and crown width as variables to establish a base model for the aboveground branch biomass and compared four basic growth functions to identify the optimal base model. Next, five age groups were introduced as dummy variables. Finally, to address the incompatibility between the biomass of individual components and total biomass in independent models, we applied the SUR method to fit the compatibility biomass model.

### 2.2 Study area overview and sample plot distribution

**Figure 2:**
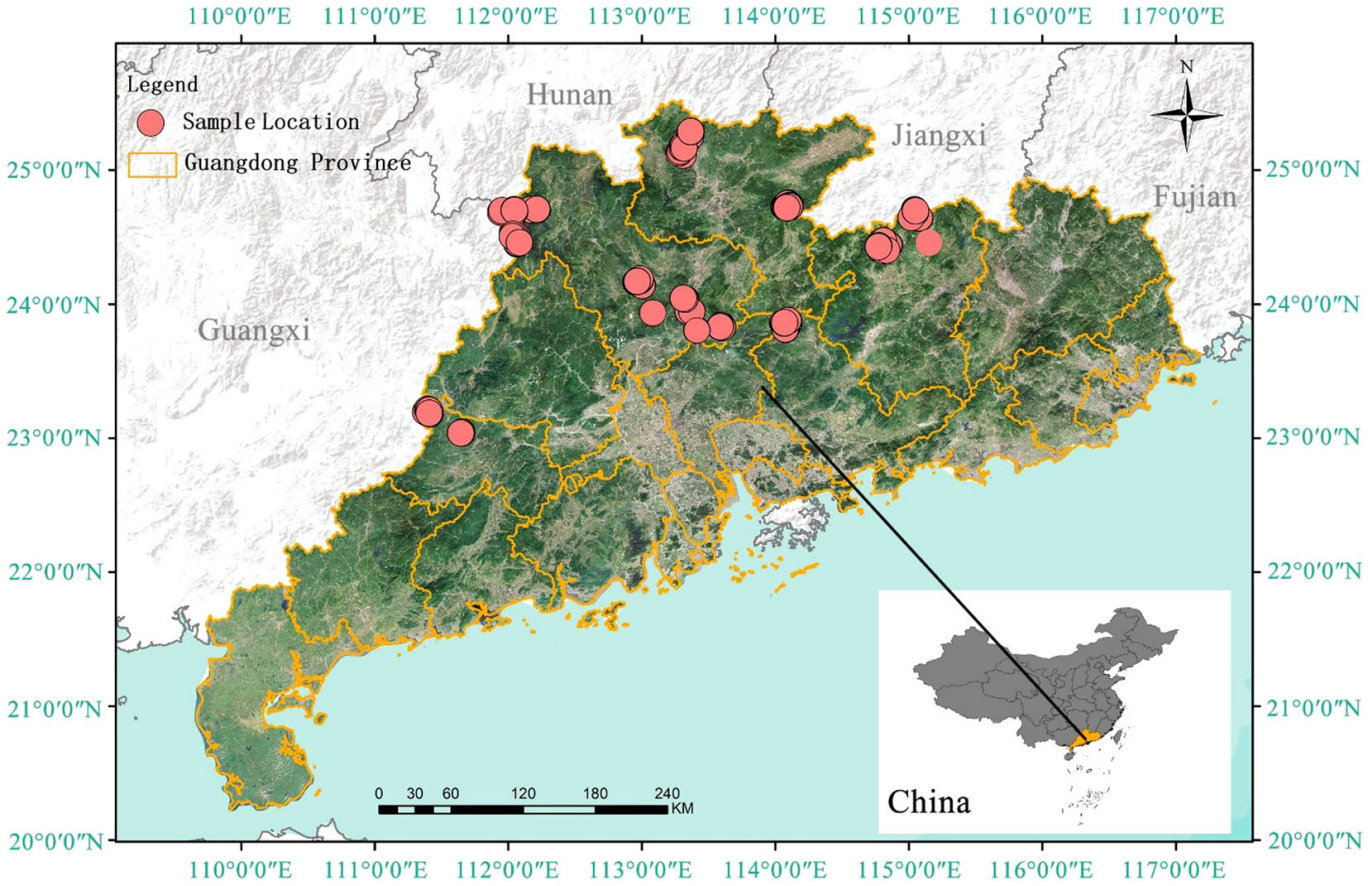
Sample point distribution map. (The locations of the sample plots are marked with red dots).

Data for this study were collected from 133 sample plots located in Lechang City, Yingde City, Heping County, Lianshan County, Longshan County, Yunan County, and Shixing County in Guangdong Province, China. To ensure spatial representativeness and reliability of these sample plots, we considered factors such as forest distribution, age groups, and accessibility during the plot selection process. Specifically, we collected data covering five distinct age groups, with each group sampled in multiple counties to encompass the entire growth cycle. For example, 29 sample plots were assigned to the youngest age group (1–10 years) with 7,481 trees, while the oldest age group (>36 years) contained 24 sample plots and 1,885 trees. Guangdong Province is situated in the southernmost part of mainland China, with geographical coordinates ranging from 109°45’ to 117°20’ east and from 20°09’ to 25°31’ north. As of 2023, the province has a forested area of approximately 10.85 million hectares, accounting for 57.1% of the total land area, with forest coverage reaching about 9.6 million hectares, which corresponds to a forest coverage rate of 53.9% (Guangdong Provincial People’s Government Portal Website, n.d). This makes it one of the key ecological protection zones in southern China. The province’s topography is mainly composed of mountains, hills, plains, and water bodies, with a general trend of higher elevations in the north and lower in the south. Guangdong is characterized by a subtropical monsoon climate, with distinct seasonal variations: warm and humid in spring, hot and rainy in summer, mild and less rainy in autumn, and cool and dry in winter. There is also significant regional climatic diversity within the province. Precipitation in the region exhibits marked monsoonal characteristics, with abundant rainfall in the summer due to the influence of the southeast monsoon, and dry, low-rainfall conditions in winter under the control of cold, dry northwesterly winds (**Figure 2**).

### 2.3 Data collection and pre-processing

#### 2.3.1 UAV LiDAR data

**Figure 3:**
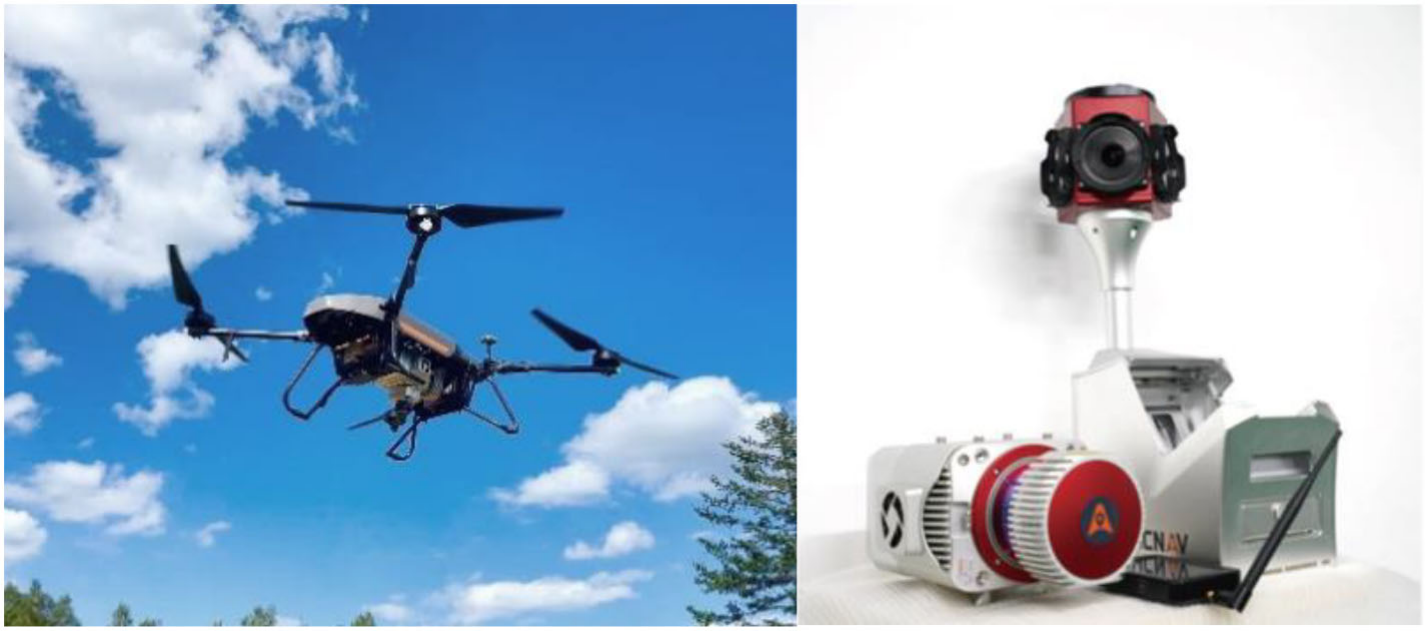
UAV Lidar system.

The UAV LiDAR data were collected in March 2024 using a HuaCe BB4 UAV equipped with an AS-1300HL LiDAR system (**Figure 3**). The scanning operation utilized a Rigel VUX-1LR scanner, which operates at a wavelength of 1500 nm, with a laser pulse duration of 3.5 ns and a divergence angle of 0.5 m rad. The LiDAR’s pulse repetition frequency was set to 50 kHz, with a maximum scanning angle of 30° and a scanning frequency of 49 Hz. A crisscross flight path was employed to ensure a lateral overlap of 50% in the point cloud data. The flight altitude was maintained at 200 m, with an average flight speed of 10 m per second, resulting in an average point cloud density of 110 points per square meter over the sample plots.

**Figure 4:**
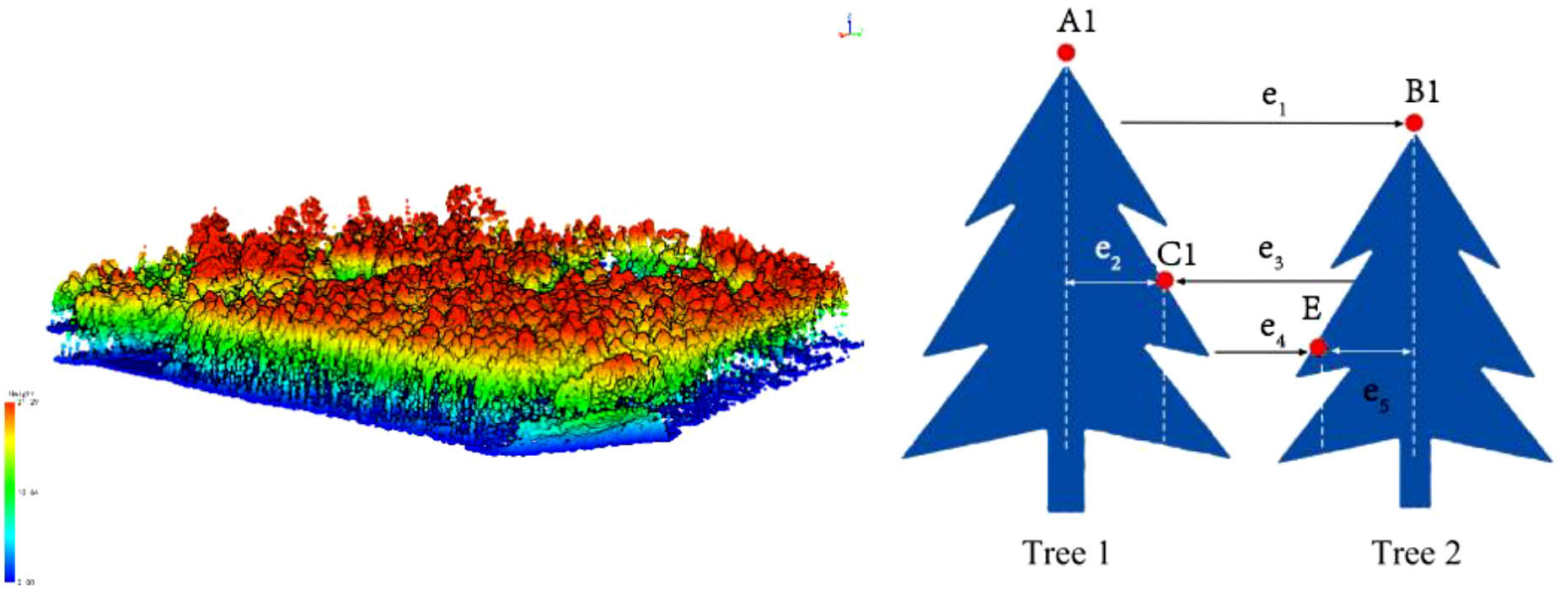
Radar-based individual tree segmentation processing.

The raw UAV LiDAR data were first visualized and interpreted using Corepore 2.0 software, followed by further interpretation using Lidar360 software. The interpreted LiDAR point clouds were then processed using the distance-based clustering algorithm to extract the radar structural features of the trees (Li et al., 2012). The spacing threshold is a parameter used in the automatic detection of individual trees to define the minimum acceptable distance between two trees. When tree canopies are very close or partially overlap, setting an appropriate spacing threshold helps to distinguish neighboring trees, preventing them from being mistakenly identified as a single tree. The minimum spacing rule is applied during individual tree segmentation in areas with high tree density, ensuring that each tree is independently identified. This is typically achieved by establishing a “buffer zone” around the detection point of each tree, within which no new trees are recognized (as illustrated in **Figure 4**). This method enabled the segmentation of individual trees within the sample plots and the calculation of key parameters such as tree height, crown width, and diameter at breast height (DBH), which are critical for estimating the biomass of individual tree components.

#### 2.3.2 Ground survey data

The field survey data for Chinese fir trees were collected simultaneously with the UAV LiDAR data in March 2024(**Table 1**). We conducted ground data surveys across 133 sample plots, each measuring 30 m × 30 m. The data were cleaned by removing deadfall, dead branches, undergrowth, litter, missing or incorrect measurements, and duplicate entries, resulting in a total of 20,836 Chinese fir (*Cunninghamia lanceolata*) trees. The survey information included species identification, DBH, tree height, height to the first branch, crown width, and growth condition. The data collection and processing strictly followed standardized field protocols to ensure accuracy as much as possible. However, given the complexity of field measurements and inherent limitations of the methods used, some natural measurement uncertainties may exist, which might be slightly amplified when handling large datasets.

**Table 1:** Field-measured biomass statistics of Chinese-fir sample trees.

		Training sample				Test sample			
AGE	Branch	Max	Min	Average	Standard deviation	Max	Min	Average	Standard deviation
AG1	Bark	13.1381	0.1096	3.3632	2.1627	13.6484	0.1078	3.3480	2.1404
	Trunk	82.1074	0.4052	17.8427	12.5648	82.2535	0.3908	17.7663	12.3915
	Branch	24.0875	0.1859	5.4190	3.5186	20.7011	0.1999	5.3753	3.5153
	Leaves	12.6573	0.2178	3.6598	1.8578	11.6341	0.3181	3.6286	1.8696
	Above ground biomass	121.232	1.032	30.285	19.8221	126.284	1.0660	30.118	19.63353
AG2	Bark	27.7597	0.1905	4.2946	2.6693	18.4729	0.1616	4.2767	2.7001
	Trunk	114.5734	0.2389	23.7724	16.2536	117.290	0.664	23.706	16.5119
	Branch	27.7597	0.1905	6.3721	4.0636	26.7768	0.1962	6.3189	4.0678
	Leaves	13.1418	0.3621	3.9614	1.9822	12.3967	0.2959	3.9307	1.9809
	Above ground biomass	167.7102	0.9027	38.4005	24.5177	172.002	1.3180	38.2320	24.7832
AG3	Bark	18.203	0.2100	5.6330	3.1345	17.3344	0.2384	5.6755	3.2327
	Trunk	119.9051	0.8125	5.6330	19.4172	119.5493	32.4140	32.4140	20.2089
	Branch	30.9941	0.3836	8.1419	4.7029	28.8945	0.3809	8.1836	4.7461
	Leaves	13.8340	0.5250	4.6920	2.1299	13.3614	0.5477	4.7083	2.1188
	Above ground biomass	172.8820	1.9910	50.5760	28.9169	170.803	2.126	50.9810	29.8144
AG4	Bark	30.2370	0.3516	9.6597	5.0867	29.1160	0.5170	9.6740	5.1307
	Trunk	202.511	1.415	58.025	33.8275	202.1930	2.2330	9.6740	34.2114
	Branch	43.9100	0.6700	13.9670	7.3899	41.6285	0.5713	13.9616	7.4742
	Leaves	20.7530	0.8990	6.9680	2.8834	17.8720	0.6087	6.9545	2.9362
	Above ground biomass	288.847	3.3350	88.6200	48.1882	276.6640	4.1920	88.7440	48.6847
AG5	Bark	28.2904	0.3132	8.9868	5.1823	32.9320	0.2010	8.9860	5.1799
	Trunk	196.453	1.1780	54.831	34.1049	219.7082	0.7777	53.8313	34.1237
	Branch	41.3550	0.5135	6.4952	7.3607	47.2662	0.3596	12.9217	7.4495
	Leaves	16.7751	0.7119	6.4952	2.8506	17.1669	0.4503	6.5330	2.9408
	Above ground biomass	279.8830	2.6970	82.1660	48.7637	316.848	1.891	82.272	48.8275

AG1, young forests, with stand ages ranging from 1 to 10 years; AG2, middle-aged forests, aged 11 to 20 years; AG3, near-mature forests, aged 21 to 25 years; AG4, mature forests, aged 26 to 35 years; AG5, over-mature forests, aged 36 years and above.

#### 2.3.3 Tree branch biomass data

**Figure 5:**
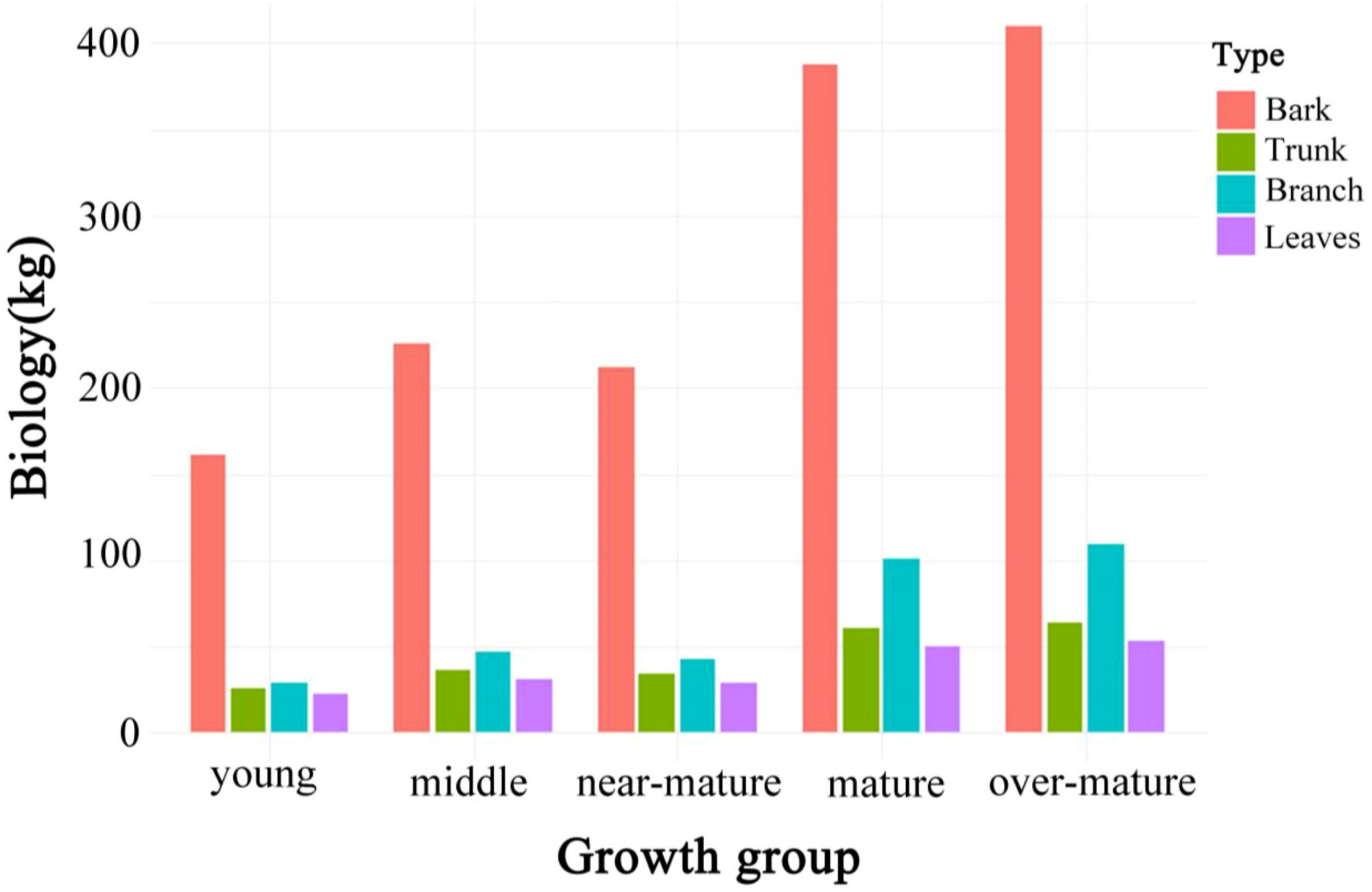
Aboveground biomass distribution across five growth stages of Chinese fir.


To analyze the differences in aboveground biomass across different developmental stages of Chinese fir, the sampled trees were first classified into five stages: young forest, middle-aged forest, near-mature forest, mature forest, and over-mature forest, based on ground survey data. Tree height, diameter at breast height (DBH), and other fundamental data were collected and recorded using consistent measurement methods. After classification, the biomass of stems, bark, branches, and leaves was calculated using the biomass equations specified in the Chinese National Standard “Biomass Models and Carbon Content Parameters for Major Tree Species” (GB/T 43648-2024). The total-tree biomass was then obtained by summing the biomass of each component, as shown in **Table 2**. Consequently, data for stem, bark, branches, leaves, and total aboveground biomass were obtained for 20,836 Chinese fir trees within the study area. These biomass data were subsequently used to model the relationship between the radar parameters derived from processed LiDAR data and the ground survey biomass measurements. **Figure 5** shows the variation trends of biomass components across different age groups, facilitating a direct comparison of growth characteristics at various developmental stages (Equations 1, 2).

**Table 2:** Summary of UAV-LiDAR-derived tree metrics.

LiDAR data	Training sample				Test sample			
	Max	Min	Average	Standard deviation	Max	Min	Average	Standard deviation
LiDAR tree height	31.6296	2.9455	13.1114	4.0801	28.771	2.7577	13.1416	4.0674
LiDAR crown diameter	16.474	0.13624	2.3081	1.4899	12.046	0.1612	2.3401	1.5169

(1) $$M_{A}={\text{a}}_{0}D^{a_{\;1}}H^{a_{\;2}}$$

(2) $$\left\{ \begin{array}{l} M_{1}=\frac{1}{1+g_{1}+g_{2}+g_{3}}\times M_{A}\\ M_{2}=\frac{{\text{g}}_{1}}{1+g_{1}+g_{2}+g_{3}}\times M_{A}\\ M_{3}=\frac{g_{2}}{1+g_{1}+g_{2}+g_{3}}\times M_{A}\\ M_{4}=\frac{g_{3}}{1+g_{1}+g_{2}+g_{3}}\times M_{A} \end{array}\right.$$

Where ***M_A_*** represents the estimated value of aboveground biomass; ***a_0_, a_1_, a_2_*** are the model parameters; is the diameter at breast height (DBH); ***H*** is the tree height; ***M_1_, M_2_, M_3_, M_4_*** represent the estimated biomass values for stem wood, bark, branches, and leaves, respectively; ***g_1_, g_2_, g_3_*** represent the ratios of the biomass of bark, branches, and leaves to the biomass of stem wood, respectively.

### 2.4 Parametric regression model

In terms of the structure of the individual tree biomass model, the model’s structure forms the basis for model construction (Zeng and Tang, 2010a; Fu et al., 2018). Using the complete growth cycle data of individual Chinese fir trees in Guangdong, this study considered four biologically meaningful theoretical tree growth models as the base models: Logistic model, Linear model, Exponential model, and Power model (**Table 3**), to fit the data. The dataset was divided into training and validation sets in a 7:3 ratio, with 70% of the data used for modeling and 30% for validation. Stepwise regression was employed to select LIDAR parameter variables, and VIF collinearity tests were conducted, excluding LIDAR feature variables with VIF > 5. LIDAR tree height (*LH*) and LIDAR crown width (*LCD*) were selected as independent variables in the model to fit the biomass components.

**Table 3:** Basic model formulae.

Kinds of models	Forms of models
Logistic Function	$BM=\frac{a}{1+bexp(-cLH-dLCD)}$
Linear Function	BM=aLH+bLCD−c
Exponential Function	BM=aexp(−bLH−cLCD)
Power Function	$BM=a^{LH^{b}LCD^{c}}$

### 2.5 Constructing dummy variable model

Dummy Variables are important for handling categorical variables. Dummy variables convert categorical variables into binary variables, allowing them to be incorporated into regression models for analysis. By introducing the above-mentioned optimal base parameter model into dummy variables, we can better understand the impact of different developmental stages of Chinese fir on biomass, thereby analyzing the effect of different age groups on total biomass. When processing the data, we converted the categorical variable “Age Group (AG)” into dummy variables. we introduced five age groups (AG) as categorical dummy variables: young stand (1–10 years), middle-aged stand (11–20 years), near-mature stand (21–25 years), mature stand (26–35 years), and over-mature stand (>36 years). The categorical variable “Age Group 1-5 (AG_1-5_)” represents five categories: young forest, middle-aged forest, near-mature forest, mature forest, and over-mature forest.

When using the age group as a dummy variable, it is necessary to convert the age group variable into a quantitative variable, usually taking the value of 0 or 1 in regression analysis. When there are n categorical attributes for the independent variable, one category is typically set as a reference, so the number of dummy variables is n-1. The formula is as follows (Equations 3, 4):


(3) $$AG_{i}=\left\{ \begin{array}{l} 1\text{ When for age group }i\\ 0\text{ If not} \end{array}\right.$$

(4) $$Y=(\begin{array}{c} \sum b_{0i}\times S_{i}) \end{array}\times (LH^{b})\times (LCD^{c})$$

where $S_{i}$ represents the dummy variables reflecting different age groups ( i = 1, 2,…, 5); $b_{oi}\;$ represents the parameters for different forest types; ***LH*** represents the Lidar tree height; ***LCD*** represents the LiDAR crown width.

### 2.6 Compatibility models

In forestry research, accurately estimating the biomass of individual tree components is crucial, especially when assessing biomass distribution and ecological functions within forest ecosystems. When both independent and dependent variables contain errors, traditional modeling methods are no longer suitable for model fitting (Wang et al., 2018b). To ensure that the predicted value of individual tree biomass equals the sum of the predicted values of its components, we must consider the additivity or compatibility among the component biomass models (Affleck and Diéguez-Aranda, 2016). Ensuring that the sum of the components is compatible with the total is of great importance for constructing biomass model systems. Additionally, additive models can account for the intrinsic relationships between components and the total, making it necessary to establish a system of simultaneous equations to achieve model compatibility.

In this study, we employed the seemingly unrelated regression (SUR) model to independently model the biomass components of aboveground biomass for Chinese fir and to jointly estimate the model parameters. This approach ensures that the stem, bark, branches, and leaves of individual trees in Chinese fir forests adhere to the principle of compatibility, thereby resolving issues of model incompatibility (Giri et al., 2019). This method not only ensures compatibility among the biomass components but also yields more optimized parameter estimates, enhancing the stability of the model (Fu et al., 2014) (Equations 5).


(5) $$\left\{ \begin{array}{l} Mds=Msg+Msp+Msz+Msy\\ Msg=(a_{1}\times AG_{1}+a_{2}\times AG_{2}+a_{3}\times AG_{3}+a_{4}\times AG_{4}+a_{5}\times AG_{5})\times (LH^{e_{1}})\times (LCD^{f_{1}})\\ Msp=(b_{1}\times AG_{1}+b_{2}\times AG_{2}+b_{3}\times AG_{3}+b_{4}\times AG_{4}+b_{5}\times AG_{5})\times (LH^{e_{2}})\times (LCD^{f_{2}})\\ Msz=(c_{1}\times AG_{1}+c_{2}\times AG_{2}+c_{3}\times AG_{3}+c_{4}\times AG_{4}+c_{5}\times AG_{5})\times (LH^{e_{3}})\times (LCD^{f_{3}})\\ Msy=(d_{1}\times AG_{1}+d_{2}\times AG_{2}+d_{3}\times AG_{3}+d_{4}\times AG_{4}+d_{5}\times AG_{5})\times (LH^{e_{4}})\times (LCD^{f_{4}}) \end{array}\right.$$

where ***AG_1-5_*** represents the different age group categories; ***a, b, c, d, e, and f*** are the model parameters; ***LH*** represents the Lidar tree height; ***LCD*** represents LiDAR crown width. ***Msg*** represents the Measured trunk biomass; ***Msp*** represents the Measured bark biomass; ***Msz*** represents the Measured branch biomass; ***Msy*** represents the Measured leaf biomass; ***Mds*** represents the Measured total aboveground biomass.

### 2.7 Model evaluation metrics

The fitting results were evaluated using four metrics: the coefficient of determination (*R²*), Root Mean Squared Error (*RMSE*), Total Relative Error (*TRE*), and Akaike Information Criterion (*AIC*). A larger *R²* indicates a higher fitting accuracy of the model, a smaller *RMSE* suggests higher precision in the model’s predictions, a smaller *TRE* indicates better predictive performance, and a smaller *AIC* represents a better model fit. The expressions are as follows (Equations 6–9):


(6) $$R^{2}=1-\sum_{i=1}^{n}{\left(y_{i}-\hat{y_{i}}\right)}^{2}/\sum_{i=1}^{n}{\left(y_{i}-\bar{y_{i}}\right)}^{2}$$

(7) $$RMSE=\sqrt{\sum_{i=1}^{n}{\left(y_{i}-\hat{y_{i}}\right)}^{2}/(n-1)}$$

(8) $$TRE=\frac{\sum \left|y_{i}-\hat{y_{i}}\right|}{\sum yi}$$

(9) AIC=2k−2ln(L)

where $y_{i}$ is the observed value of the dependent variable; $\hat{y_{i}}$ is the predicted value of the dependent variable by the model; $\bar{y_{i}}$ is the mean of the observed values; ***n*** is the number of observations; ***k*** is the number of parameters in the model. ***L*** is the maximum likelihood of the model.

## 3 Results and analysis

### 3.1 Optimal model selection

**Figure 6:**
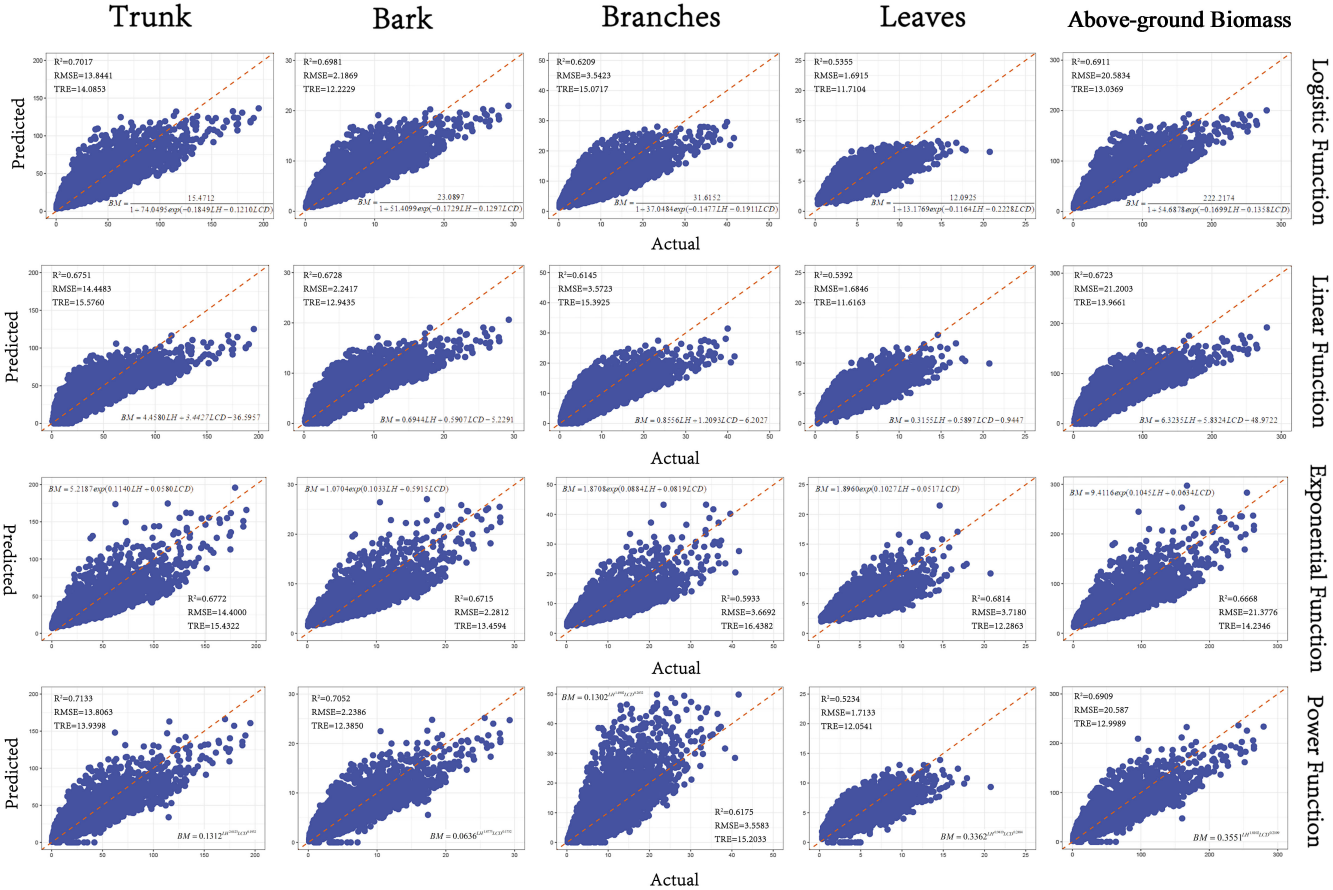
Goodness-of-fit plots of the base models.(The x-axis represents the actual values, and the y-axis represents the predicted values. The *R²* is the coefficient of determination, *RMSE* is Root Mean Squared Error, and *TRE* is Total Relative Error).

Based on the goodness-of-fit plot results of the 4 basic models (**Figure 6**), the Power model was determined to be the optimal model through comprehensive consideration of *AIC*, *R²*, *RMSE*, and *TRE*. Since the Logistic model and the Power Function model had similar validation parameters, we chose the Power Function model as the optimal model due to its fewer parameters.

The Power Function Model exhibited the highest *R²* values, particularly for stem and bark biomass, effectively capturing allometric growth relationships but lacking adaptability to different growth stages. In terms of the fitting performance of the aboveground biomass component models, the *R²* values for all components except leaves and branches were above 0.7, with the fitting accuracy order being stem > bark > branches > leaves (**Table 4**).

**Table 4:** Detailed parameters and fitting accuracy of the 4 types of base models.

Model form	Type	Model parameter						Training sample			Test sample		
		*a*	*b*	*c*	*d*	AIC	BIC	*R^2^*	*RMSE*	*TRE*	*R^2^*	*RMSE*	*TRE*
Logistic Function	Bark	152.4712	74.0495	0.1849	0.1210	118516	118554.	0.6968	14.0616	14.7311	0.7017	13.8441	14.0853
	*SE*	3.0908	1.6807	0.0025	0.0036								
	Trunk	23.0897	51.4099	0.1729	0.1297	64684	64721	0.6938	2.2213	12.2229	0.6981	2.1869	12.2229
	*SE*	0.4412	1.0812	0.0024	0.0037								
	Branch	31.6152	37.0484	0.1477	0.1911	78855	78893	0.6172	3.6106	15.9090	0.6209	3.5423	15.0717
	*SE*	0.6479	0.8338	0.0024	0.0048								
	Leaves	12.0925	13.1769	0.1164	0.2228	57095	57133	0.5346	1.7125	12.1670	0.5355	1.6915	11.7104
	*SE*	0.2203	0.2831	0.0025	0.0062								
	Above ground biomass	222.2174	54.6878	0.1699	0.1358	130137	130175	0.6862	20.9428	13.6838	0.6911	20.5834	13.0369
	*SE*	4.5191	1.1468	0.0023	0.0037								
Linear Function	Bark	4.4580	3.4427	-36.5957		119805	119835	0.6687	14.6976	16.3161	0.6751	14.4483	15.5760
	*SE*	0.0326	0.0891	0.4069									
	Trunk	0.6944	0.5907	-5.2291		65453	65483	0.6772	2.2808	13.5626	0.6728	2.2417	12.9435
	*SE*	0.0051	0.0138	0.0633									
	Branch	0.8556	1.2093	-6.2027		79155	79185	0.6092	3.6481	16.2959	0.6145	3.5723	15.3925
	*SE*	0.0081	0.0221	0.1011									
	Leaves	0.3155	0.5897	-0.9447		57008	57038	0.5373	1.7075	12.0880	0.5392	1.6846	11.6163
	*SE*	0.0038	0.0104	0.0476									
	Above ground biomass	6.3235	5.8324	-48.9722		131043	131073	0.6660	21.6046	14.6914	0.6723	21.2003	13.9661
	*SE*	0.0479	0.1311	0.5986									
Exponential Function	Bark	5.2187	-0.1140	-0.0580		119897	119927	0.6666	14.7439	16.4360	0.6772	14.4000	15.4322
	*SE*	0.0672	0.0007	0.0016									
	Trunk	1.0704	-0.1033	-0.5915		66102	66133	0.6624	2.3322	14.2688	0.6715	2.2812	13.4594
	*SE*	0.0126	0.0007	0.0016									
	Branch	1.8708	-0.0884	-0.0819		80057	80087	0.5843	3.7627	17.5174	0.5933	3.6692	16.4382
	*SE*	0.0241	0.0008	0.0017									
	Leaves	1.8960	-0.1027	-0.0517		80235	80266	0.6736	3.7858	12.9121	0.6814	3.7180	12.2863
	*SE*	0.0178	0.0007	0.0016									
	Above ground biomass	9.4116	-0.1045	-0.0634		131449	131479	0.6566	21.9076	15.1694	0.6668	21.3776	14.2346
	*SE*	0.1147	0.0008	0.0016									
Power Function	Bark	0.1312	2.0123	0.1932		118537	118567	0.6963	14.0724	14.7571	0.7133	13.8063	13.9398
	*SE*	0.0051	0.0136	0.0051									
	Trunk	0.0636	1.8773	0.1752		83695	83726	0.6994	2.2056	13.0076	0.7052	2.2386	12.3850
	*SE*	0.0015	0.0124	0.0047									
	Branch	0.1302	1.4902	0.2832		78998	79028	0.6134	3.6286	16.0935	0.6175	3.5583	15.2033
	*SE*	0.0003	0.0191	0.0066									
	Leaves	0.3362	0.9419	0.2484		57404	57434	0.5246	1.7308	12.4618	0.5234	1.7133	12.0541
	*SE*	0.0102	0.0112	0.0049									
	Above ground biomass	0.3551	1.8102	0.2109		130208	130238	0.6846	20.9950	13.7616	0.6909	20.5870	12.9989
	*SE*	0.0129	0.0129	0.0049									

*a*,*b*,*c*,*d*, the model parameters; *AIC*, Akaike Information Criterion; *R^2^*, Coeffcient Of Determination; *RMSE*, Root Mean Square Error; *TRE*, Total Relative Error; *SE*, Standard Error.

**Table 4** includes standard errors (*SE*) for key parameters. Lower *SE* values typically indicate more stable parameter estimates, whereas higher *SE* values suggest potential variability or limitations in data coverage for those biomass components. These *SE* values help illustrate how reliably each model parameter can be estimated from the available data, thereby offering insight into the overall robustness of each model’s predictions (Equations 10–14).


(10) $$Msg=0.1312^{LH^{2.0123}LCD^{0.1932}}$$

(11) $$Msp=0.0636^{LH^{1.8773}LCD^{0.1752}}$$

(12) $$Msz=0.1302^{LH^{1.4902}LCD^{0.2832}}$$

(13) $$Msy=0.3362^{LH^{0.9419}LCD^{0.2484}}$$

(14) $$Mds=0.3551^{LH^{1.8102}LCD^{0.2109}}$$

where ***Msg*** represents the stem biomass; ***Msz*** represents the branch biomass; ***Msy*** represents the leaf biomass; ***LH*** stands for radar tree height; and ***LCD*** stands for radar crown width.

### 3.2 Dummy variables model

In this study, the qualitative factors of the five age groups—young forest, middle-aged forest, near-mature forest, mature forest, and over-mature forest—were converted into quantitative factors using the method of dummy variables. These were then introduced into the optimal models for the biomass components of Chinese fir, including stem, bark, branches, and leaves. By analyzing Chinese fir at different developmental stages, we can better understand the impact of these stages on biomass.

When comparing the dummy variable model with the previously determined optimal base models, we found that the *RMSE* of the model decreased by an average of 3%. Among the components, the reduction was most significant for bark, which decreased by 4.2%, followed by branches with a reduction of 3.1%. The reductions for stem and leaves were the smallest, both at 2.4%. The *R²* increased by an average of 2.6%, with the most notable improvements in the precision of leaves and branches, which increased by 3% and 2.8%, respectively. The remaining components showed an average improvement in precision of 2% (**Table 5**). This highlights the importance of considering growth stages in biomass estimation, making the dummy variable model more adaptable to complex mixed forests with coexisting age groups. This indicates that incorporating dummy variables into the base models allows for a better fit of the data and can effectively model the biomass components of individual trees across different age groups.

**Table 5:** Dummy variable parameters and fitting accuracy.

Model form	Type	Model parameter							Training sample			Test sample		
		*a_1_*	*a_2_*	*a_3_*	*a_4_*	*a_5_*	*b*	*c*	*R^2^*	*RMSE*	*TRE*	*R^2^*	*RMSE*	*TRE*
Power function	Bark	0.2221	0.2034	0.1929	0.2582	0.2524	1.8152	0.1936	0.7193	13.5287	13.4880	0.7198	13.4717	13.0373
	*SE*	0.0094	0.0097	0.0093	0.0129	0.0123	0.0178	0.0049						
	Trunk	0.0682	0.0627	0.0598	0.0781	0.0763	1.6104	0.1939	0.7147	2.1441	11.7994	0.7140	2.1287	11.4484
	*SE*	0.0027	0.0026	0.0027	0.0036	0.0035	0.0164	0.0046						
	Branch	0.2290	0.2088	0.2024	0.2756	0.2635	1.2731	0.2870	0.6434	3.4849	14.6613	0.6403	3.4507	14.1326
	*SE*	0.0099	0.0098	0.0101	0.0141	0.0132	0.0181	0.0055						
	Leaves	0.4902	0.4537	0.4476	0.5695	0.5447	0.7961	0.2508	0.5533	1.6777	11.6213	0.5461	1.6720	11.3978
	*SE*	0.0176	0.0175	0.0187	0.0246	0.0231	0.0151	0.0048						
	Above ground biomass	0.5919	0.5405	0.5156	0.6873	0.6685	1.6189	0.2122	0.7095	20.1484	12.5377	0.7091	19.9734	12.1172
	*SE*	0.0241	0.0238	0.0241	0.0329	0.0314	0.0169	0.0048						

*a_1_*, *a_2,_ a_3,_ a_4,_ a_5,_ b* ,*c* the model parameters; *R^2^*, Coeffcient Of Determination; *RMSE*, Root Mean Square Error; *TRE*, Total Relative Error; *SE*, Standard Error.

In addition, **Table 5** provides the *SE* of key parameters for each age group, illustrating potential uncertainties in parameter estimates. Generally, smaller *SE* values indicate more stable parameter estimates, whereas larger *SE* values may suggest data limitations or higher variability in certain developmental stages. Overall, incorporating dummy variables into the base models yields a better data fit and can effectively capture the biomass components of individual trees across different age groups.

### 3.3 Compatible biomass modeling

**Figure 7:**
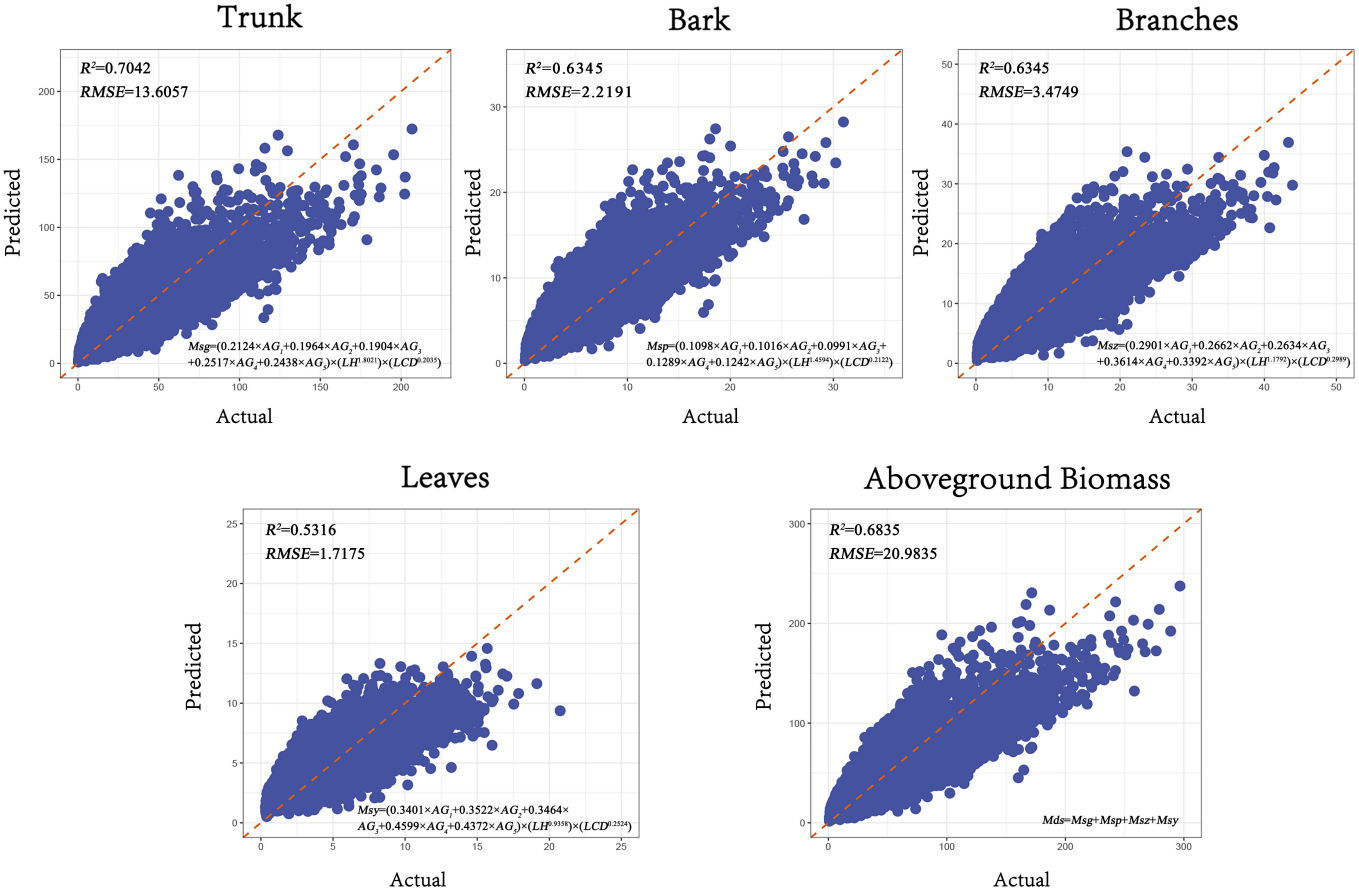
Goodness-of-fit plots of the SUR model. (The x-axis represents the actual values, and the y-axis represents the predicted values. The *R²* is the coefficient of determination, *RMSE* is Root Mean Squared Error).

The compatibility of the aboveground biomass model is achieved by jointly modeling the biomass components of the tree, including stem, bark, branches, and leaves, and simultaneously solving the model parameters. This approach addresses the inconsistency between the component biomass and total biomass that can occur with independent models. The SUR (Seemingly Unrelated Regression) method was used to fit the additive biomass model, and the results showed that the model had a good fit (**Figure 7**).

As shown in **Table 6**, compared to the dummy variable model, the accuracy of the *R²* values decreased by an average of 2.5%. The Root Mean Squared Error *RMSE* for the biomass components—stem, bark, branches, and leaves—and the total aboveground biomass are 13.6057, 2.2191, 3.4749, 1.7175, and 20.9835, respectively. Compared to the dummy variable model, the *RMSE* increased by 2.6%. This reduction in predictive accuracy may be due to the constraints imposed by the simultaneous equation system to maintain consistency among biomass components. Minimizing residuals for one component might introduce slight errors in others. Additionally, the joint estimation of multiple equations may amplify estimation variance due to correlations among error terms, further affecting model performance.

**Table 6:** SUR model form and fitting accuracy.

Forms of sub-modeling	Model fitting accuracy	
	*R^2^*	RMSE
$Msg=(0.2124AG_{1}+0.1964AG_{2}+0.1904AG_{3}+0.2517AG_{4}+0.2438AG_{5})\times (LH^{1.8021})\times (LCD^{0.2035})$	0.7042	13.6057
$Msp=(0.1098AG_{1}+0.1016AG_{2}+0.0991AG_{3}+0.1289AG_{4}+0.1242AG_{5})\times (LH^{1.4594})\times (LCD^{0.2122})$	0.6927	2.2191
$Msz=(0.2901AG_{1}+0.2662AG_{2}+0.2634AG_{3}+0.3614AG_{4}+0.3392AG_{5})\times (LH^{1.1792})\times (LCD^{0.2989})$	0.6345	3.4749
$Msy=(0.3401AG_{1}+0.3522AG_{2}+0.3464AG_{3}+0.4599AG_{4}+0.4372AG_{5})\times (LH^{0.9358})\times (LCD^{0.2524})$	0.5316	1.7175
Mds=Msg+Msp+Msz+Msy	0.6835	20.9835

*R^2^*, Coeffcient Of Determination; *RMSE*, Root Mean Square Error.

## 4 Discussion

### 4.1 Main findings and patterns

Chinese fir (*Cunninghamia lanceolata*), as one of the main tree species in Guangdong and the entire southern region of China, plays a significant role in ecosystem services, enhancing forest carbon sequestration, climate regulation, and economic benefits (Zeng et al., 2017). With the increasing pressures of climate change and ecological environmental stress, the technological methods for acquiring and analyzing forestry data have become increasingly important. The timely and effective acquisition of biomass, carbon storage, and other forest survey data has become a focal point in forest science research (He et al., 2013; Chen et al., 2023). In this study, we utilized UAV LiDAR technology to collect data on 20,836 Chinese fir trees across 133 sample plots in Guangdong Province and calculated the biomass for each tree component. Following the initial selection of the optimal base model, we further introduced age groups as dummy variables to model the different age stages of the Chinese fir. Finally, we applied the Seemingly Unrelated Regression (SUR) model to ensure the compatibility between radar tree height (LH) and radar crown width (LCD) with the biomass of individual tree components, while also maintaining the additivity of the biomass components (Chen et al., 2023).

### 4.2 Model accuracy and performance

#### 4.2.1 Applicability and limitations of base models

Establishing universal or regional relative growth equations for forest biomass has long been a goal in forestry and ecology (Brown and Lugo, 1984; Fang et al., 2001; Jenkins et al., 2003; Lehtonen et al., 2004; Guo et al., 2010). The combination of multiple models and the application of statistical models can better predict forest biomass; however, due to factors such as habitat, climate, and geography, even the same tree species may exhibit significant biomass differences in different regions (Zeng et al., 2011; Forrester et al., 2017). Moreover, the characteristics of biomass accumulation and distribution of Chinese fir vary significantly across different developmental stages, indicating that simple base models may not accurately reflect the dynamic changes in Chinese fir biomass, potentially leading to errors and uncertainties in model equations (Deng D, et al., 2023). Previous studies (Chen et al., 2016; Zhang D. et al., 2019) mainly relied on traditional ground measurement methods, which were often time-consuming, labor-intensive, and limited in data volume. In contrast, this study innovatively adopted UAV LiDAR technology, greatly improving the efficiency and quality of data collection.

#### 4.2.2 Importance of differentiating growth stages

During the development of biomass models for Chinese fir plantations, previous studies found that incorporating age groups as dummy variables significantly improved model accuracy compared to traditional methods (e.g., Guo et al., 2016; Shen et al., 2016), consistent with our findings. However, these studies often relied on limited ground measurements, restricting coverage of the full growth cycle and spatial applicability.

This study identified significant stage-specific differences in the biomass allocation of Chinese fir across five growth stages. During the young and middle-aged stages, trees prioritize the development of photosynthetic organs to enhance competitiveness and rapidly accumulate carbon. At this stage, the biomass proportion of leaves and branches is higher, facilitating rapid canopy expansion and improving photosynthetic efficiency, which strengthens competitiveness under resource limitations (Canham et al., 1996). The higher biomass of leaves and branches also increases canopy coverage and enhances species diversity (Eriksson et al., 2006). As trees enter the mature and over-mature stages, biomass shifts toward the stem and bark (Gerrish, 1990), particularly in the mature stage, where stem biomass increases significantly, indicating that resources are primarily used to enhance structural stability and long-term carbon storage (Molina-Valero et al., 2021). Increased bark biomass improves resistance to pests and environmental stress, enhancing survival rates (Cernusak and Cheesman, 2015). Moreover, the substantial biomass in the stem strengthens mechanical support and boosts the forest’s carbon sequestration capacity (Castaño-Santamaría et al., 2013). Therefore, forest management should focus on promoting leaf and branch growth during the early stages to enhance growth vigor and ecological adaptability, while in the later stages, efforts should shift toward supporting stem and bark development to improve resilience and long-term carbon storage (Wernick and Kauppi, 2022).

#### 4.2.3 Advantages of the dummy variable model with age groups

In contrast, our study innovatively utilized remote sensing data, enabling comprehensive coverage of all growth stages and substantially enhancing the model’s applicability and generalization across broader spatial scales. Therefore, we introduced age groups as categorical dummy variables based on the optimal base model to simulate and model Chinese fir at different developmental stages. The study demonstrated that the dummy variable model, when incorporating age group indicators, outperformed the base model in statistical metrics such as *R²*, *TRE*, and *RMSE*, highlighting the limitations of base models in establishing large-scale biomass models for Chinese fir (Jianfeng and Jian, 2021). By introducing age groups as dummy variables into the model, it more comprehensively reflects the biomass differences across different developmental stages of Chinese fir, thereby enhancing the model’s fit. Compared to the optimal base model, the dummy variable model, after incorporating different developmental stages, showed an average *R²* increase of 3.2%, indicating that developmental stages have a significant impact on the accumulation and distribution of Chinese fir biomass. Therefore, adding age groups as dummy variables can improve the predictive accuracy of the biomass components, a finding that is consistent with Shen et al. (2019) (Lv and Duan, 2024). Their research also showed that incorporating age groups as dummy variables into the model significantly improved the model’s fit, with the best predictive performance observed for stem biomass, followed by branches and bark, while the predictive accuracy for leaf biomass was the lowest (Chen et al., 2023). The conclusions drawn from this study align with these findings, suggesting that the poor predictive performance for leaf biomass may be due to the relatively small proportion of leaves in the total aboveground biomass.

Even though the fitting results of the model should be kept within a reasonable expected range to ensure its effectiveness and reliability are not affected by bias (Zhang et al., 2013), in practice, the biomass of individual tree components often exhibits significant variation. This variation may stem from issues encountered during the data fitting process. In this study, through the modeling analysis of the age groups of 20,836 Chinese fir trees in Guangdong Province, we found that the ranking of aboveground biomass was: stem > bark > branches > leaves. The biomass of bark and leaves in young forests was relatively low, which suggests that in practical applications, different model types should be established according to age groups to improve the accuracy of biomass estimation results. While the dummy variable model effectively handles specific data and characteristics of different components, relying solely on the dummy variable model to process Chinese fir biomass data may still have limitations. This is because the dummy variable model, when operating independently, may not fully account for the intrinsic relationships and interactions among the different components of aboveground biomass within individual trees (Wang et al., 2008; Fu et al., 2012). Ignoring these interactions can lead to biased or inconsistent prediction results, particularly when dealing with larger datasets.

#### 4.2.4 Compatibility and accuracy trade-off in the SUR model

The Seemingly Unrelated Regression (SUR) model integrates these independent models, ensuring that the mathematical relationships and inherent correlations between total aboveground biomass and its components are maintained, specifically the additive or compatible relationship among the different components (Fu et al., 2014). In the past, many reported biomass equations lacked additivity or compatibility, with independent equations established for each component. However, when scholars compared the fitting accuracy of additive biomass models with non-additive biomass models, they found that using non-additive methods to construct models could lead to significant discrepancies between the sum of the biomass of individual tree components and the total biomass of the tree. If these models are applied in practice, they could result in errors (Parresol, 1999; Fu et al., 2016; Dong et al., 2018). The SUR model ensures compatibility among biomass components, resolving summation inconsistencies at the cost of a slight decrease in predictive accuracy (*R²* decreased by 2.5%, *RMSE* increased by 2.6%). This decline may result from the constraints imposed by the system of simultaneous equations, which can introduce errors in other components when minimizing the residuals for one component. Additionally, the joint estimation of multiple equations in the SUR model may amplify variance due to correlations between error terms, increasing computational complexity and affecting model performance (Poudel and Temesgen, 2016; Nord-Larsen et al., 2017; Zhao et al., 2019).In contrast, the dummy variable model may not fully utilize the potential correlations between the equations (Zellner, 1962).

### 4.3 Limitations

Although the models developed in this study performed well for estimating Chinese fir biomass in subtropical regions, their performance may vary under different environmental conditions or for other tree species. The models were established under humid and stable subtropical climate conditions with abundant rainfall; therefore, in drier regions, trees may grow more slowly and allocate biomass differently among stems, branches, and leaves, which could reduce model accuracy. Similarly, at higher altitudes, factors such as wind exposure and shallow soil may affect tree growth patterns, requiring adjustments to the model parameters. Additionally, different tree species have distinct growth strategies. For example, broadleaf trees typically allocate more biomass to branches and leaves, while coniferous trees tend to concentrate biomass in the stem (Zhang et al., 2020). Therefore, if these models are applied to other tree species or different regions, parameter adjustments may be necessary to reflect local growth patterns better. Furthermore, due to limitations in field survey time, manpower, and resources, the sampling sites could not cover all target regions, which may affect the representativeness and applicability of the models. Also, the data used in this study were mainly collected from specific geographic and climatic conditions, which may limit the model’s generalizability to other environments or species. Future research could enhance model applicability and predictive accuracy by expanding the sampling coverage and incorporating data from different environmental conditions and tree species.

## 5 Conclusions

This study employed both the dummy variable model and the SUR model to develop aboveground biomass component models for Chinese fir. Introducing the age-group dummy variable increased the mean coefficient of determination (R²) from 0.69 to 0.71, an improvement of 2.6%, and reduced the total-biomass RMSE by 3%. For branch and bark biomass, R² rose by 3.1% and 4.2%, while their RMSE values fell by 3.1% and 4.2%, respectively. The SUR model ensured consistency between component and total biomass, achieving an overall R² of 0.684; its RMSE was 2.6% higher than that of the dummy-variable model but offered greater stability in managing inter-component relationships. Moreover, the integration of UAV LiDAR data with ground measurements provided robust technical support for precise biomass estimation, laying a solid foundation for long-term monitoring and sustainable management of Chinese-fir ecosystems. Moreover, the integration of UAV LiDAR technology with ground-based manual measurement data provided robust technical support for the precise estimation of Chinese fir biomass, establishing a solid foundation for the long-term monitoring and sustainable management of the Chinese fir ecosystem.

## Data Availability

The datasets presented in this article are not readily available because the data is restricted due to confidentiality issues; therefore, the dataset cannot be shared. Requests to access the datasets should be directed to Liyong Fu, fuly@ifrit.ac.cn.
